# Supplementary material for: Mellow and Thick Taste of Pu−Erh Ripe Tea Based on Chemical Properties by Sensory−Directed Flavor Analysis
Source: Foods. 2022 Jul 31;11(15):2285. doi: 10.3390/foods11152285 (PMC9368183; doi:10.3390/foods11152285)
Supplement: Supplementary file 1 [file foods-11-02285-s001.zip › foods-1831740-supplementary.pdf]

Supplementary materials

Mellow and Thick Taste of Pu-erh Ripe Tea Based on Chemical Properties by Sensory-directed Flavor Analysis

Sihan Deng [1, 2], Xinru Zhou [1], Haiyu Dong [1], Yong-Quan Xu [2], Ying Gao [2 *], Baijuan Wang [1,3,4], Xiaohui Liu [1,3,4 *]

1. College of Tea Science, Yunnan Agriculture University, Kunming, 650201, China.
2. Tea Research Institute Chinese Academy of Agricultural Sciences, National Engineering Research Center for Tea Processing, Key Laboratory of Biology, Genetics and Breeding of Special Economic Animals and Plants, Ministry of Agriculture and Rural Affairs, 9 South Meiling Road, Hangzhou 310008, China.
3. Yunnan organic tea industry Intelligent Engineering Research Center, Kunming, 650201, China.
4. Key Laboratory of intelligent organic tea garden construction in Universities of Yunnan Province, Kunming, 650201, China.

Corresponding Authors

[*] Xiaohui Liu. Email: 2014042@ynau.edu.cn.

[*] Ying Gao. Email: yinggao@tricaas.com.

Table S1. Sensory evaluation of SGT and PRT initial infusions.

Taste attributes	SGT	PRT
Bitterness	4.90±0.34	1.75±0.29**
Astringency	3.01±0.45	1.35±0.31**
Mellow and thick	2.68±0.54	4.45±0.17**
Sweetness	1.50±0.24	2.88±0.25**
Overall taste	6.73±0.46	7.75±0.29*

SGT: sun-dried green tea; PRT: pu-erh ripe tea. Different lowercase letters in the same row indicate significant differences between data. ** indicates $p < 0.01$; * indicates $p < 0.05$

Table S2. Contributions of PRT, RL, and FL to the mellow and thick taste.

Taste attributes	PRT	RL	FL
Bitterness	1.75±0.29^{a}	1.50±0.58^{a}	1.18±0.35^{a}
Astringency	1.35±0.31^{a}	1.25±0.50^{a}	1.13±0.25^{a}
Mellow and thick	4.45±0.13^{b}	6.68±0.24^{a}	1.38±0.25^{c}
Sweetness	2.88±0.25^{a}	3.38±0.48^{a}	3.28±0.32^{a}
Overall taste	7.75±0.29^{a}	8.13±0.30^{a}	6.13±1.03^{b}

PRT: pu-erh ripe tea; RL: rejection liquid, FL: filtration liquid. Different lowercase letters in the same row indicate significant differences between data ($p < 0.05$).
